# Silicon and Mechanisms of Plant Resistance to Insect Pests

**DOI:** 10.3390/plants7020033

**Published:** 2018-04-13

**Authors:** Fadi Alhousari, Maria Greger

**Affiliations:** Department of Ecology, Environment and Plant Science, Stockholm University, 10691 Stockholm, Sweden; maria.greger@su.se

**Keywords:** silicon, plant resistance, insects, HIPVs, physical defence, induced defence

## Abstract

This paper reviews the most recent progress in exploring silicon-mediated resistance to herbivorous insects and the mechanisms involved. The aim is to determine whether any mechanism seems more common than the others as well as whether the mechanisms are more pronounced in silicon-accumulating than non-silicon-accumulating species or in monocots than eudicots. Two types of mechanisms counter insect pest attacks: physical or mechanical barriers and biochemical/molecular mechanisms (in which Si can upregulate and prime plant defence pathways against insects). Although most studies have examined high Si accumulators, both accumulators and non-accumulators of silicon as well as monocots and eudicots display similar Si defence mechanisms against insects.

## 1. Introduction

Arthropod pests are biotic stressors, attacking plants above and below ground and eventually reducing yield quantity and quality [1]. Plants counteract insect attacks both directly and indirectly. Many of these defences are regulated by signalling pathways in which phytohormones have central roles. Direct defences associated with host morphological traits such as trichomes, wax and cell wall lignification affect insect feeding behaviour and performance. These plant characteristics constitute physical or mechanical feeding barriers as the first line of defence. The second line of defence comprises secondary metabolites (e.g., phenols and lignin, which affect insect growth and development), with various enzymes, such as polyphenol oxidase (PPO), phenylalanine ammonia lyase (PAL) and peroxidase (POD), being involved in their synthesis. Indirect defences are mediated by host plant volatiles or by herbivore-induced plant volatiles (HIPVs) released in response to insect feeding. HIPVs, modulated by the JA pathway, promote biological control by attracting predators and parasitoids of the insect pests [2,3,4,5,6,7,8]. Both direct and indirect responses to insect attacks contribute to plant resistance and may be constitutively present or induced [4]. 

In addition, exploiting plant resistance can represent an economically and ecologically efficient approach to integrated pest management (IPM). One way to improve effective resistance is to supplement with silicon (Si). Si is an important element in plant nutrition and is the most common element, after oxygen, on earth. Silicic acid, that is, Si(OH)_4_, is the bioavailable form of silicon in soil solution that is taken up by plant roots [9,10]. Si is translocated through the xylem to the shoots where it condenses into polymerized silica gel [11]. According to their ability to accumulate Si, plants are classified as high (10–15%), medium (1–3%) and non-(<1% Si dry mass, dm) Si accumulators [12]. High Si accumulators include wetland grasses (e.g., rice, bamboo and sugar cane) and medium accumulators terrestrial grasses (e.g., wheat), while low accumulators are commonly eudicots. 

It is now well established that Si enhances plant resistance and reduces plant damage caused by pathogens, insect pests and non-insect pests through the mediation and upregulation of both resistance mechanisms that are constitutive (i.e., irrespective of insect presence) and induced (i.e., in response to insect attack) [13,14]. To date, a range of examples documents the ability of Si to enhance the resistance of both monocotyledonous crops and numerous dicot plant species to insect pests of diverse feeding guilds belonging to Lepidoptera [8,15,16], Hemiptera [17], Homoptera [18], Diptera [19], Thysanoptera and Coleoptera [20] as well as to non-insect pests [21,22]. 

**Figure 1:**
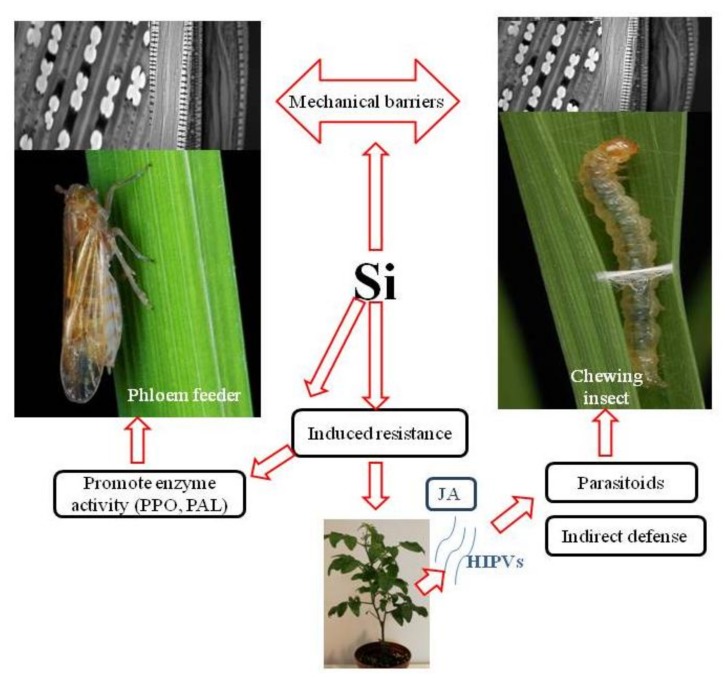
Silicon mediated mechanisms of plant resistance to insect pests. (PPO) polyphenol oxidase, (PAL) phenylalanine ammonia lyase, (HIPVs) herbivore-induced plant volatiles, (JA) jasmonate phytohormone.

Si deposition patterns within plant tissues led to the hypothesis of mechanical or physical barriers to insect feeding, as silica makes plant tissues difficult for insects to efficiently chew, penetrate and digest. In addition, silica’s beneficial roles in plant physiology, regulation of defence-related enzymes, plant hormone signalling and alteration of plant volatile blends elucidate the association of Si with biochemical/molecular defence mechanisms ([15,20,23,24,25]; Figure 1). 

In this review, we describe the mode of action of Si in plant resistance mechanisms and highlight how Si bolsters plant defences against insect pests of different feeding guilds in both mono- and dicots.

## 2. Formation of Physical Barriers to Insects

**Figure 2:**
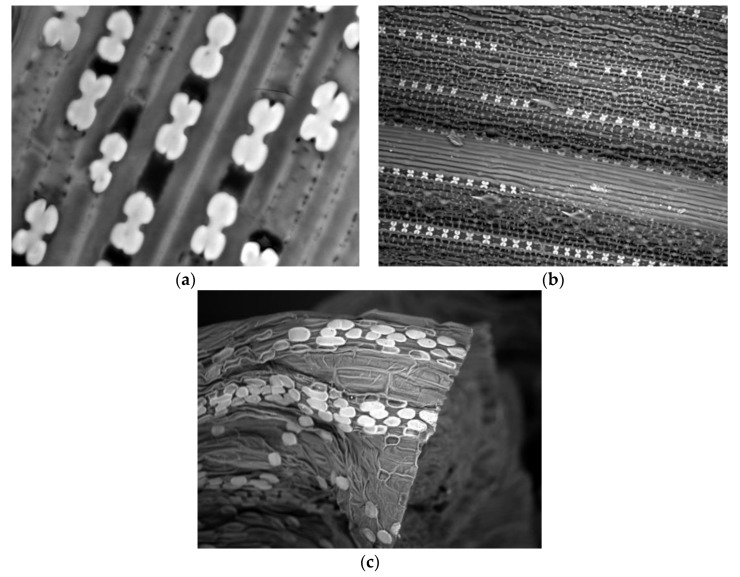
Scanning electron micrographs of maize (**a**); rice (**b**); and wheat (**c**) sheath surfaces showing silica cell form and deposition.

The physical resistance mechanism was first proposed with reference to fungal diseases in eudicots and then generalized to monocots (see mini-review by Fauteux et al. [23]). The bioavailable Si absorbed by plants generally strengthens direct and indirect plant resistance to insect pests via the deposition of SiO_2_ as biogenic opals (phytoliths), primarily in the epidermal cells of leaves, stems and roots [14]. Silicon is deposited as a 2.5-µm-thick layer just beneath the cuticle layer (0.1 µm thick), forming a silicon–cuticle double layer in rice leaf blades ([26]; Figure 2). Consequently, phytoliths promote cell-wall strengthening. The abrasiveness of silicified leaves and other plant tissues associated with protection, storage, support and strengthening leads to the increased irreversible wear of mouthparts when insects are feeding, therefore deterring chewing insects. Mouthpart wear due to Si treatment can vary according to feeding habit. For instance, *Spodoptera exempta* larvae fed a silica-rich diet displayed increased mandible wear [27]. In contrast, no damage was observed in the incisor teeth of the mandibles, imaged by scanning electron microscopy (SEM), of leaf miner (*Tuta absoluta*) larvae fed Si-treated tomato leaves. In other words, this could be due to the specific feeding strategy, since leaf miners feed on soft tissues between epidermal cell layers [4,28]. 

Moreover, a high Si level could influence the availability of other nutrients in plants, such as nitrogen, inducing insects to consume greater quantities of high-Si-treated plants. In addition, a high silica content in plant tissue reduces its digestibility and palatability, consequently slowing the insect growth rate [15,27,29]. 

The insect midgut epithelium plays an important role in food digestion and conversion to nutrients by digestive enzymes; moreover, it is a site for insecticide detoxification [30]. Si could damage the ultrastructure of the midgut epithelium, mainly through detachment of epithelial cells from the basement membrane as observed in larvae of the leaf miner *Tuta absoluta* fed Si-treated leaves of tomato (an Si excluder) [28]. This negatively affects the nutrient absorption and growth rate. It could also prevent insects from developing resistance to pesticides and could increase the efficacy of chemical controls combined with Si. 

On the other hand, Si is involved in toughening plant tissues, acting indirectly by delaying insect penetration of host tissues and thus increasing the duration of insect exposure to natural enemies, adverse environmental conditions and chemical controls. In sugarcane, Si accumulated in the stem epidermal tissue of the internode and root band increased the resistance to *Eldana saccharina* by reducing larval stalk penetration [15,31].

Silica could also protect the resources in the chlorenchyma cells of grasses against locusts (*Schistocerca gregaria*) by reducing mechanical breakdown of the leaf. In addition, Si can also help increase grasses resistance by reducing chlorophyll released after grinding and retained more after passing through the gut of locusts [32].

In plants of coffee, a dicot, Si may also enhance a plant morphological trait that confers resistance to insect feeding through the formation of a thicker wax layer on the abaxial surface of coffee seedlings [33].

Furthermore, the arrangement and distribution of silicified microstructures, together with their pattern and location in plant tissues, were considered more effective at conferring resistance than was their actual Si content, effectively delaying plant penetration by insects and thereby decreasing plant susceptibility to herbivore insect damage. Si amendment increases the content in leaf sheaths and the histological parameters of silica cells. Such as rows of silica cells per mm^2^, number of silica cells per 1-mm row and area of silica cells, consequently maximizes the physical barrier to insect pests approximately tenfold in rice [7,31,34]. 

SEM investigation of Si-treated rice plants revealed ladder-like structures of dumbbell-shaped silica and Si-enriched trichomes. These microstructures in rice impart strength to the plant and serve as a mechanical barrier against stem borers and planthoppers ([11,35]; Figure 2). The sharp Si-enriched trichomes mechanically affect the insects, impeding their movement and settlement and possibly negatively affecting their oviposition preference and feeding rate [36]. In addition, glandular trichomes function as deterrents by secreting secondary metabolites (e.g., flavonoids, terpenoids and alkaloids) that can be poisonous and repellent to many insect pests, thus increasing resistance [37].

Likewise, our SEM observations revealed various forms of silica cells, which are butterfly shaped in maize and rice leaves and oval in wheat (Figure 2). These different shapes and distributions of silicified microstructures could be attributed to the Si concentration and plant growth stage. Si deposition could shift from small cells to bulliform cells and trichomes as the Si content increased in rice plants [35]. Furthermore, Si cells differentiation and accumulation expected to be regulated by JA [24].

Si additionally has physiological, biological and behavioural consequences in the insects. It is possible to affect the development of insect pests, their population intensity and feeding behaviour. Larval survival and pupation rate of the rice leaf folder *Cnaphalocrocis medinalis* Guenée (Lepidoptera: Pyralidae) were significantly reduced by feeding on rice plants supplemented with Si [8]. Extended larval development means that instars lack the food quality and food conversion efficiencies. These elements enhance the resistance in a rice variety (Taichung Native 1, TN1) susceptible to the rice leaf folder *C. medinalis* Guenée. Si amendment in rice is equally responsible for physiological and behavioural implications in the phloem-feeding insects by reducing the fertility, honeydew excretion quantity and settled insect number of the brown planthopper *Nilaparvata lugens* Stål. (Homoptera: Delphacidae). Moreover, a high Si addition could affect the sucking behaviour by prolonging the stylet pathway and time needed to achieve the first phloem puncture and shortening the durations of phloem puncture and phloem ingestion [18,38].

Among other biological parameters, the rate of fecundity was the most affected in *Spodoptera frugiperda* female derived from caterpillars feeding on corn diet treated with Si [39]. 

Not only shoots but also roots can defend against insect attacks. Interestingly, high root Si concentrations can effectively reduce the feeding and relative growth rate performance of the sugarcane root-feeding insect, the greyback canegrub (*Dermolepida albohirtum*) [40,41]. 

To sum up, based on the above studies and findings, it can be concluded that Si confers resistance to plants species against insect pests by forming physical barriers (in both mono- and dicots) and eventually impacts on insect feeding behaviour and performance. 

## 3. Silicon-Mediated Induced Resistance to Insects

The use of plant resistance inducers is considered an environmentally friendly strategy to efficiently decrease insect pest populations. In addition to acting as a mechanical barrier, Si can reduce pest damage by enhancing the induced chemical defences of plants following insect attack. Silicon acts as an abiotic elicitor of systemic stress signals, mediated by phytohormone pathways, leading to the efficient synthesis of defensive compounds [23]. Plant defences are complex and can vary according to the feeding strategy of the insect pests [42].

Each plant attacker has its own signal signature. The common phytohormones salicylic acid (SA), jasmonic acid (JA) and ethylene play primary roles in orchestrating plant defence responses [43]. JA is suggested to regulate defences against both cell-content-feeding and tissue-chewing insects [44,45]. Defence against phloem-feeding insects is regulated by both SA and JA signals [46]. Interestingly, evidence for the strong interaction between Si and JA against insects is accumulating [24,47], this being considered a possible mechanism by which Si enhances resistance to insect pests. Moreover, Si-induced resistance could also be expressed by priming the host plant to defend itself against insect pests attack [20,48,49,50]. Priming is a process of sensitizing and preparing the plant’s defence responses to be faster and stronger to future herbivorous insect threats [6,24]. 

Next, we will focus on recent studies of the role of Si in induced plant defence responses to chewing and phloem-feeding insect pests.

### 3.1. Si and Chewing Insect Pests

Plant secondary metabolites play a vital role in plant interactions with insects and their natural enemies. In addition, plant volatile emissions can be constitutive or can be induced in response to stresses. Regardless of the emission mode, volatiles are involved in defence reactions triggered by herbivores [51]. 

In tritrophic systems, chemical compounds are emitted by plants in reaction to insect-induced damage in the form of HIPVs. These compounds can act either as direct attractants or repellents of insects and thus may be used as host-finding cues by entomophagous predators and parasitoids of insect pests ([52,53]; Figure 1). 

Si may trigger different plant species to emit, amplify, and/or alter HIPVs. In response to feeding by the rice leaf folder (*C. medinalis*), a wild-type rice plant supplied with Si mounts a strong indirect defence based on HIPV production. Among which are hexanal 2-ethyl, α-bergamotene, β-sesquiophellandrene and cedrol, produced in significantly smaller amounts in infested Si-treated plants [47]. 

These changed HIPV profiles then significantly enhanced the attraction of adult females of the parasitoids *Trathala flavo-orbitalis* and *Microplitis* to the Si-treated plants attacked by *C. medinalis*. The signalling pathways that allow rice plants to mount resistance against the chewing insect *C. medinalis* are JA dependent [47]. To elaborate, Si and JA linked strongly to different components of rice defensive system. This can be expressed in increasing the levels of transcripts encoding defence genes, the activities of defence-related enzymes (PPO, POD and trypsin protease inhibitor), in addition to HIPVs alteration [24].

Under both laboratory and semi-field conditions, Si-treated plants attracted significantly more of the predator *Dicranolaius bellulus* to cucumber plants (a medium Si-accumulator dicot) infested with *Helicoverpa armigera* [54].

Another well-established example of this phenomenon is in *Vitis vinifera* L., a dicot and Si non-accumulator. A positive correlation was observed between plant tissue Si content and attraction of the predator *D. bellulus* to grapevines infested with *Epiphyas postvittana*. Moreover, seven volatile compounds emitted in *Phalaenoides glycinae*-infested grapevines were identified. One of them, n-heptadecane, was released in significant amounts only by Si-fertilized grapevines [55].

The above-mentioned literature revealed that Si application could notably alter HIPVs in both mono and dicots plant species while sharing the same response against chewing insect pests.

### 3.2. Si and Phloem Feeders 

Insect phloem feeding can be inhibited at three stages: before food ingestion, during ingestion (via the activity of salivary enzymes), or after digestion and food absorption. Electrical penetration graphs (EPGs) allowed monitoring of the behavioural responses of insects during probing and feeding and exploration of interference with probing by chemical or physical factors within the plant tissues and of the localization of resistance within plant tissues [56]. 

Based on EPG findings, reduction of both duration of phloem ingestion and proportion of the brown planthopper (*N. lugens*) individuals ingesting phloem were observed on rice amended with Si. Silicon-induced resistance to *N. lugens* is associated with increased accumulation of callose. Callose deposition in the sieve tubes blocks the mass flow of phloem and prevents phloem sap leakage following feeding puncture [38,57]. 

Si may further involve in biochemical and physiological changes that triggered by H_2_O_2_ in rice plant tissue upon *N. lugens* attack. Si amendment could obviously alleviate the stress resulting from *N. lugens* by slowing the increase of malondialdehyde (MDA) concentrations, the physiological index of plants under stress. Moreover, Si plays a role in scavenging the reactive oxygen species (ROS) by priming the activities of antioxidant enzymes. Immediately after *N. lugens* attack, The PPO and PAL activities trigger and catalyse the oxidation of phenols to quinines. It can reduce the palatability of plant tissues and eventually restrict insect development [4,7]. 

Similarly, treating wheat plants with silicon could negatively affect the feeding behaviour and population increase rate of the greenbug *Schizaphis graminum* Rondani (Hemiptera: Aphididae). Suppressing the percentage of *S. graminum* reached the phloem ingestion phase indicates that Si-induced resistance possibly localized at the phloem level. The Si-induced mechanism in wheat plants could be explained by increasing the activities of POD, PPO and PAL. The POD is involved in plant defence via lignification, suberization and production of ROS and quinones, which exhibit antibiotic properties [58,59].

Si-induced resistance has also been reported in eudicots as well as monocots. In cucumbers, Si may induce the synthesis of defence chemicals, reducing the preference of *Bemisia tabaci* (Gennadius) (Hemiptera: Aleyrodidae) for oviposition, expanding the insects’ developmental period and increasing nymphal mortality [60]. 

Briefly stated, the above studies clearly show that Si supplementations can induce several plant defence responses, to phloem-feeding insect pests, by modulating the plant antioxidant defence systems and secondary metabolites.

## 4. Si-Induced Resistance below Ground

Like aboveground plant parts, belowground portions of plants are also face threats, namely, from root-feeding insects. Interestingly, the attack of aboveground plant shoots by insects can also result in root responses defending against root feeders. Induced defences mediated by JA signalling have been found to improve rice resistance to the rice water weevil (*Lissorhoptrus oryzophilus*), whose larvae feed on rice roots under flooded conditions [61]. Accordingly, the interaction between both constitutive and Si-induced resistance could strongly enhance plant resistance and reduce damage caused by root-feeding insects. 

Below ground, the larvae of *Diabrotica speciosa* (Coleoptera: Chrysomelidae) damage plant roots and create holes in the tubers of the potato (*Solanum tuberosum* L.), whereas the adults consume the leaves. Foliar applications of silicic acid, an inducer of plant resistance, increased plant protection against defoliators and decreased tuber damage, reducing the number of holes in the tubers of treated plants. This reduction in tuber attack was correlated with the reduced leaf damage in the plants treated with silicic acid [62]. 

A recent study demonstrated that Si nanoparticles (SiNPs) may induce defence responses in the root system [25]. The authors demonstrated that SiNPs increased the lignification of the root cell wall in the dicot fenugreek, *Trigonella foenum-graecum* (Fabaceae), together with increasing the expression of the root defensive gene (*tfgd* 1) [25,63]. 

Moreover, root-applied Si optimizes the mechanical characteristics of rapeseed by increasing the root diameter, breaking strength and expression levels of the key genes related to stem lignin biosynthesis [64]. 

## 5. Summary and Future Research

As described here, Si has a central role in boosting plants’ direct and indirect defences against many insect pests via two Si-based mechanisms: strengthened physical or mechanical barriers and biochemical/molecular mechanisms that induce plant defence responses. The relevant studies have been performed in various plant species, often using insects with diverse feeding strategies. Taken together, we draw the overall conclusion that plants employ both Si-based resistance mechanisms synergistically rather than singly, relying on combined physical, chemical and biochemical mechanisms to reduce damage by insect pests. 

For example, the brown planthopper is affected by both the physical barrier of silica cells and by the induced resistance mediated by Si in rice as a model high Si accumulator (Table 1). 

It also seems that Si-mediated mechanisms act similarly in plants both below and above ground, as Si induces lignin accumulation in the roots of both sugarcane (a monocot) [41] and oilseed rape (a eudicot) [37], increasing toughness and, eventually, resistance to insect attack [69]. Though the accumulation of Si differs among plant species, they likely display similar Si defence mechanisms against insects. Similarly, monocot and eudicot species seem to respond similarly to insect attack through similar Si-mediated mechanical and biochemical mechanisms. Accordingly, we predict that as-yet-untested insect pests may be affected in the same way as tested species.

Generally, chewing insects and phloem-feeding insects (e.g., whitefly and aphids) induce distinct plant responses to attack. Chewing herbivores have stronger inductive effects than do sucking ones [70,71]. For example, compared with the chewing caterpillar *Spodoptera exigua*, the phloem feeder *Bemisia tabaci* did not induce the emission of HIPVs in *Gossypium hirsutum* [70,72]. Similarly, *Spodoptera littoralis* induced HIPV emissions whereas the aphid *Rhopalosiphum maidis* induced no measurable emissions even after heavy infestations in the monocot *Zea mays* [73]. Regardless of the effect magnitudes, Si affects both direct and indirect plant defences against both chewing and sucking insects, leading to similar impacts on biological parameters such as development time, immature survival and rate of population increase. Moreover, Han et al. [8] and Lang et al. [7] reported similar chemical defence responses via activation of the defensive enzyme that protects plants from stress in Si-amended rice infested with *C. medinalis* and *N. lugens*, respectively. However, there is little information on the role of Si mediated resistance through HIPVs induction against phloem feeding insects. 

Chewing insects are more susceptible to Si physical barriers than are phloem feeders, as the latter may be able to avoid the phytoliths but we cannot dismiss the possibility that plant tissue injury resulting from the feeding itself may trigger the battery of Si-induced plant responses. 

Further studies are therefore required to explore: Si-mediated resistance to insect pests in non-Si-accumulating plant species, both mechanical and biochemical mechanisms of insect pest resistance and the correlation between constitutive and induced resistance in which Si plays a role. Silicon, with all its remarkable protective plant defence effects, could be an eco-friendly alternative to conventional pesticides in IPM in agriculture.

## Figures and Tables

**Table 1:** Si-mediated plant resistance mechanisms and defensive responses reported in the literature.

Crop	Insect Species	Resistance Mechanism	Reference
Grasses *Lolium perenne* L. and *Festuca ovina* L.	Locust *Schistocerca gregaria*	Mechanical protection of resources in chlorenchyma cells	[32]
Rice	Rice leaf folder *Cnaphalocrocis medinalis*	Reduced insect food quality and food conversion efficiencies; priming defence-related enzymes	[8,65]
Rice	Rice leaf folder *Cnaphalocrocis medinalis*	Induced defence based on HIPV production	[47]
Rice	Asiatic rice borer *Chilo suppressalis* Walker	Impeded stalk penetration and prolonged penetration duration by early instar larvae	[16]
Rice	Brown planthopper *Nilaparvata lugens* Stål.	Modulation of callose deposition	[66]
Rice	Brown planthopper *Nilaparvata lugens* Stål.	Antibiotic and xenobiotic effects targeting insect physiological functions	[18]
Rice	Brown planthopper *Nilaparvata lugens* Stål.	Physical barrier and induced chemical defences	[7,38]
Corn	Armyworm *Spodoptera frugiperda*	Affected biological parameters (fecundity of females)	[39]
Sunflower	Sunflower caterpillar *Chlosyne lacinia saundersii*	Affected feeding behaviour due to leaf palatability	[67]
Potato	Beetle *Diabrotica speciosa*	Negatively affected oviposition and feeding behaviour	[62]
Wheat	Green bug *Schizaphis graminum* Rondani	Induced defences affecting preference and suppressing population increase	[58,59]
Cucumber	Whitefly *Bemisia tabaci*	Induced defences (synthesis of defensive chemicals) reducing the whitefly population	[60]
Bean	Whitefly *Bemisia tabaci*	Negatively affected oviposition preference development of nymphs	[68]
Sugarcane	Greyback canegrub *Dermolepida albohirtum*	Increased lignin accumulation	[41]
